# Non-O157 Shiga toxin-producing Escherichia coli infections in Europe.

**DOI:** 10.3201/eid0304.970425

**Published:** 1997

**Authors:** A Caprioli, A E Tozzi, G Rizzoni, H Karch


### Non-O157 Shiga Toxin–Producing *Escherichia coli* Infections in Europe

**To the Editor:** Shiga toxin–producing *Escherichia coli* (STEC) infections are an important cause of severe human disease. Although most infections are caused by strains of serogroup O157, STEC pathogenic to humans may belong to other serogroups usually referred to as non-O157 STEC.

Recently, Tarr et al. (1) and Acheson et al. (2) described infections attributable to STEC O103 and expressed concern that non-O157 STEC may pose an underestimated threat to public health in the United States. In fact, non-O157 STEC is often overlooked in clinical microbiology laboratories because the toxigenic phenotype is not exploited to identify such pathogens. Rather, most laboratories use sorbitol MacConkey agar and serotyping (which cannot detect most non-O157 STEC) to identify *E. coli* O157:H7.

Since the end of the 1980s, non-O157 STEC infections have caused as many as 10% to 30% of sporadic cases of hemolytic uremic syndrome (HUS) in Germany (3), Italy (4), and the United Kingdom (5). Moreover, HUS outbreaks have been associated with STEC O111:H- in Italy (6) and France (7).

During 1996, we observed a sudden increase in infections attributable to STEC O103 and O26 in Germany and Italy. In our laboratory in Germany, *E. coli* O103:H2 was not identified among 345 non-O157 STEC isolated between 1985 and 1995 but represented 12 (18.2%) of 66 of the non-O157 STEC isolated during 1996. HUS developed in two infected patients.

Among cases reported to Italy's nationwide HUS surveillance system from 1988 to 1995, evidence of infection with STEC O103 or O26 was found in two (1.5%) and nine (6.6%) of 135 cases, respectively. Since 1996, infection with STEC O103 and O26 has been diagnosed in three (11%) and nine (33%) of 27 HUS cases, respectively.

These observations indicate that identification of non-O157 STEC should be considered by clinical laboratories. Immunoenzymatic tests (based on either toxin antibodies or receptors) that detect Shiga toxins produced by fecal bacterial isolates or present in stool specimens are now available (8,9). Use of these tests should be considered in analyzing the stools of patients with HUS, bloody diarrhea, or painful nonbloody diarrhea, if classic microbiologic analysis fails to yield *E. coli* O157:H7 or another standard enteric pathogen, such as Campylobacter, Salmonella, or Shigella.

The sudden appearance or increase of rare non-O157 STEC in our populations is worrisome. Most non-O157 STEC, as well as the sorbitol fermenting O157:H- strains (10) associated with HUS in several European countries, would be missed by laboratories using standard microbiologic detection methods, such as sorbitol MacConkey agar screening. Because of the considerable clinical and epidemiologic urgency, clinical microbiologists and physicians should seek out these such pathogens in appropriate clinical situations.

**Alfredo Caprioli,* Alberto E. Tozzi,* Gianfranco Rizzoni,† and Helge Karch‡**
*Istituto Superiore di Sanità, Rome, Italy;
†Ospedale Pediatrico Bambino Gesù, Rome, Italy;
‡Universitat Wuerzburg, Germany

### References

1. Tarr PI, Fouser LS, Stapleton AE, Wilson RA, Kim HH, Vary JC, Clausen CR. Hemolytic-uremic syndrome in a six-year-old girl after a urinary tract infection with Shiga-toxin-producing *Escherichia coli* O103:H2. N Engl J Med 1996;335:635-8.
2. Acheson DWK, Wolf LE, Park CH. *Escherichia coli* and the hemolytic-uremic syndrome. N Engl J Med 1997;336:515.
3. Bitzan M, Moebius E, Ludwig K, Muller-Wiefel DE, Heesemann J, Karch H. High incidence of serum antibodies to *Escherichia coli* O157 lipopolysaccharide in children with the hemolytic uremic syndrome. J Pediatr 1991;119:380-5.
4. Caprioli A, Luzzi I, Rosmini F, Pasquini P, Cirrincione R, Gianviti A, et al. Hemolytic-uremic syndrome and vero-cytotoxin-producing *Escherichia coli* infection in Italy. J Infect Dis 1992;166:154-8.
5. Kleanthous H, Smith HR, Scotland SM, Gross RJ, Rowe B, Taylor CM, Milford DV. Haemolytic-uraemic syndromes in the British Isles, 1985-8: association with verocytotoxin producing *Escherichia coli*. Part 2: microbiological aspects. Arch Dis Child 1990;65:722-7.
6. Caprioli A, Luzzi I, Rosmini F, Resti C, Edefonti A, Perfumo F, et al. Communitywide outbreak of hemolytic-uremic syndrome associated with non-O157 verocytotoxin-producing *Escherichia coli*. J Infect Dis 1994;169:208-11.
7. Boudaillez B, Berquin P, Mariani-Kurkdjian P, Bef D, Cuvelier B, Capek J, et al. Possible person-to-person transmission of *Escherichia coli* O111-associated hemolytic uremic syndrome. Pediatr Nephrol 1997;11:36-9.
8. Kehl KS, Havens P, Behnke CE, Acheson DWK. Evaluation of the premier EHEC assay for detection of Shiga-toxin-producing *Escherichia coli*. J Clin Microbiol 1997;35:2051-4.

9. Basta M, Karmali M, Lingwood C. Sensitive receptor-specified enzyme-linked immunosorbent assay for *Escherichia coli* verocytotoxin. J Clin Microbiol 1989;27:1617-22.
10. Gunzer F, Bohm H, Russmann H, Bitzan M, Aleksic S, Karch H. Molecular detection of sorbitol-fermenting *Escherichia coli* O157 in patients with hemolytic-uremic syndrome. J Clin Microbiol 1992;30:1807-10.

### The Taxonomy of *Cyclospora*

**To the Editor:** In the article by N.J. Pieniazek and B.L. Herwaldt (1) on the rRNA gene of *Cyclospora cayetanensis*, the authors suggest that *Cyclospora* should be placed in the genus *Eimeria* because the rRNA genes of the two genera have similar sequences. The article refers to Norman D. Levine's chapter on the Apicomplexa in the Illustrated Guide to the Protozoa (2). Regrettably, the authors failed to read the whole chapter and to recognize that the initial characteristics for placing the oocyst of a coccidium in its proper genus are the number of sporocysts and then the number of sporozoites in each sporocyst. The genus *Eimeria* has four sporocysts and two sporozoites in each sporocyst. The genus *Cyclospora* has two sporocysts, each of which has two sporozoites.

The original taxonomists (3) of *C. cayetanensis* recognized that it should be placed in the taxonomic family Eimeriidae, close to *Eimeria*, but they adhered to the traditional designation for genera of coccidia. Pieniazek and Herwaldt should be cognizant of the rules of zoologic nomenclature as well as the fact that certain morphologic characteristics of protists have served us well for many decades and continue to be useful. There are serious consequences to changing the classification of an organism, and it should not be thought that one can make such a change casually. I encourage the editors of Emerging Infectious Diseases to seek the advice of those who understand what should be done with respect to the classification and nomenclature of organisms.

**William C. Marquardt**
Colorado State University, Fort Collins, Colorado, USA

### References

1. Pieniazek NJ, Herwaldt BL. Reevaluating the molecular taxonomy: Is human-associated *Cyclospora* a mammalian *Eimeria* species? Emerg Infect Dis 1997;3:381-3.
2. Levine ND. Apicomplexa. In: Lee JJ, Hutner SH, Boyce EC, editors. An illustrated guide to the protozoa. Lawrence (KS): Society of Protozoologists; 1985. p.322-74.
3. Ortega YR, Gilman RH, Steling CR. A new coccidian parasite (Apicomplexa:Eimeriidae) from humans. J Parasitol 1994;80:625-9.

**Reply to W.C. Marquardt:** Dr. Marquardt's advocacy for reliance on morphologic characteristics even if phylogenetic data become available that lead to a different conclusion runs counter to that expressed in an article he coauthored, which supports the importance of molecular data (1). The introduction of that paper states the following:

"Early systematists relied largely on light microscopic structures and life cycle patterns to separate protozoa taxonomically.... Apicomplexans display enormous variations in life cycle patterns, physiology, cytology, and biochemistry. There is no consensus on which characteristics should be relied upon to infer phylogenetic relationships. Developmental and ultrastructural features have been used to infer evolutionary relationships among representative genera in the class Sporozoea. However, comparisons of phenotypic characters are qualitative and lack objective quantitative assessment to infer genetic relationships. Sequence similarities between proteins or genes which share a common evolutionary history can be used to infer quantitative phylogenetic relationships. The small subunit (16S-like) rRNAs and their coding regions are especially useful for estimating the extent of genetic relatedness over broad evolutionary ranges."

That paper concludes with the statement that "ribosomal RNA sequence analyses of other apicomplexans are required in order to test the validity of relationships inferred from structures and life cycle patterns." Similarly, we concluded our paper as follows: "Reports based on morphologic features alone may suffer from poor resolution of features needed for classification of closely related

